# Induced Ruminal Lactic Acidosis in Sheep Treated with Various Remedial Agents in Libo Kemkem Districts, Northwest Ethiopia

**DOI:** 10.1155/2024/5595475

**Published:** 2024-07-11

**Authors:** Balemual Abebaw, Achenef Melaku, Shimelis Dagnachew

**Affiliations:** ^1^ Department of Veterinary Science, P.O. Box: 272Debre Tabor University, Debre Tabor, Ethiopia; ^2^ Department of Veterinary Pharmacology, P.O. Box: 196University of Gondar, Gondar, Ethiopia; ^3^ Department of Para-clinical Studies, P.O. Box: 196University of Gondar, Gondar, Ethiopia

## Abstract

**Background:**

Grain overload is a ruminant metabolic disorder associated with overingestion or a sudden change to rapidly fermentable concentrate feeds.

**Objective:**

A randomized clinical trial was carried out in Libo Kemkem districts to investigate vital signs, ruminal fluid, and hematological changes in sheep related to grain overload and to assess the treatment efficacy of various remedial agents in Farta sheep breeds.

**Methods:**

All sheep were selected by using the simple random process, and categorized into four groups of eight animals groups I, II, and III in which each sheep was given 80 g of wheat flour per kg of body weight then baking soda, Magnalax, and digestive powder were treated successively, but group IV was the negative control group.

**Results:**

The current clinical trial explained that all acidotic sheep had statistically significant (*p* < 0.0002) lower body temperature, rumen motility, protozoa activity, and ruminal fluid pH with 38.48 ± 0.20, 0.5 ± 0.89, 6 (75%), and 5.37 ± 0.34 mean value successively. Nevertheless, there were significant (*p* < 0.0059) increments in hematological variables including total red blood cell, total white blood cell, packed cell volume, and hemoglobin estimation with 14.05 ± 1.27, 12.71 ± 0.99, 40.78 ± 5.24, and 14.78 ± 1.83 mean value, respectively, before treatment in acidotic sheep. There were also vital sign increments including pulse rate, respiratory rate, skin turgor, and capillary refill time with 123.5 ± 27.1, 73.75 ± 12.71, 3 ± 1.78, and 3.37 ± 1.77 mean values, respectively, before medical treatment in acidotic sheep. Digestive powder was the first drug of choice, followed by Magnalax, and baking soda was ranked at the end based on clinical recovery.

**Conclusion:**

The treatment effectiveness illustrated that all treatments could cure the grain overload. Nevertheless, digestive powder is the drug of choice based on the clinical and systemic recovery of the sheep. In addition to this, sheep should be introduced gradually to concentrate rations over 2–3 weeks with a mixture of concentrate in the milled feed containing roughage.

## 1. Introduction

Ruminal lactic acidosis is a metabolic disorder defined by decreased blood pH and bicarbonate, caused by overproduction of ruminal D-lactate [1]. It results from the high production of lactic acid in the rumen after ingestion of large quantities of readily digestible carbohydrates, particularly grain. Wheat, barley, and corn are the most readily digestible grains; oats are less digestible [2]. Rumen overload in sheep occurs due to the ingestion of grains, which helps the development of *Streptococcus bovis* bacteria [3]. Morbidity rate and mortality rate vary based on the type of grain, the dose of grain eaten, and body condition score [2].

The clinical signs of grain overload include reduction of appetite, fluid and electrolyte loss due to diarrhea, ataxia, grinding of teeth, grunting, and abdominal distension [4]. The treatment of ruminal acidosis depends on the degree of severity of the case. A variety of treatments can be given like antibiotics to reduce the growth of Gram-positive bacteria and fluid and electrolytes to maintain circulating blood volume [5]. Sodium bicarbonate, magnesium hydroxide, and digestive powder are remedial agents that correct the disturbed blood pH to a normal level. Digestive powder is an appetizer, which induces reticulorumen motility. It consists of different components: paracetamol, glucose monohydrate, sodium propionate, and sodium hydrogen carbonate. Sodium chloride oral solution is given to treat fluid and electrolyte loss due to diarrhea [6]. Sodium bicarbonate or magnesium hydroxide is applied to alkalinize the rumen pH (power of hydration). Fiber supplementation is given for the prevention of ruminal acidosis. Sheep should be introduced gradually to concentrate rations over 2–3 weeks, beginning with a mixture of ≤50% concentrate in the milled feed containing roughage [7].

Sheep and goat production in Ethiopia contributes a main role to the GDP (gross domestic product) of the country [8]. Nevertheless, grain overload has a significant economic impact by resulting in delayed marketing, condemnation of entire carcasses, reduced weight gain, reduction of appetite, and death of sheep [9].

Various remedial agents have different scientific names but are used for the correction of disturbed parameters related to ruminal lactic acidosis. It includes magnesium hydroxide or Magnalax, sodium bicarbonate or baking soda, and digestive powder, which are used to correct the disturbed ruminal and systemic acidosis to the normal required level of pH value in animals. Therefore, they have similar characteristics as the alkalinizer of ruminal acidosis [10]. However, differentiation among remedial agents for the treatment of ruminal and systemic acidosis due to gain overload is essential for accurate and rapid therapy of this disease. There has been no study about acute carbohydrate engorgement in sheep and its treatment efficacy of various remedial agents in Farta sheep breeds in Libo Kemkem districts, North West Ethiopia Therefore, the objective of this study is to investigate the treatment efficacy of various remedial agents of induced carbohydrate engorgement in Farta sheep breeds.

## 2. Materials and Methods

### 2.1. Study Area

**Figure 1:**
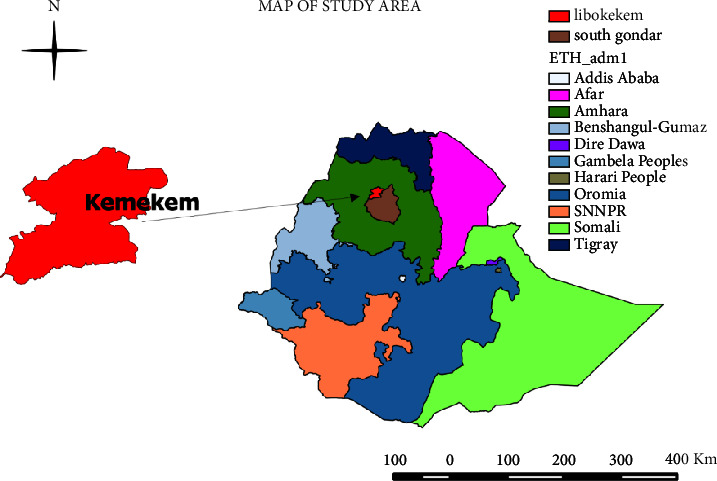
Map of the study area (we created the map with QGIS version 3.10.2 software) (https://qgis.org/en/site/forusers/download.html).

The study was carried out in the Libo Kemkem district. It is placed in the south Gondar zone of the Amhara regional state (Figure 1). The district has a latitude and longitude of 12°07′N 37°47′E and an elevation of 1975 meters above sea level. The animal population consists of 124,607 cattle, 157,811 small ruminants, 27,329 equines, 199,950 poultry, and 21,381 honeybee hives [11].

### 2.2. Study Design

A randomized clinical trial was carried out in Libo Kemkem districts.

### 2.3. Study Population

Indigenous sheep breeds (Farta sheep) were brought from the market with a variety of ages and sexes selected in the Libo Kemkem district.

### 2.4. Study Animals

A total of 32 Farta sheep breeds were selected by using the simple random method with a mean body weight of 28 kg (range 25–34 kg). The sheep were given straw before and during the clinical trial. Sheep was off feed for twenty-four hours before the start of the clinical trial. During the clinical trial, each group was penned separately in their room. For this experiment, 32 sheep were randomly divided into 4 groups of eight animals in each group. On day zero, all sheep were offered 80 g of wheat flour per kg of body weight. The flour was offered as a suspension in warm water by using a stomach tube [12].

### 2.5. Study Methodology

#### 2.5.1. General Examination

All groups were observed three times in day associated with their posture, appetite, rumination, and diarrhea. General examination with clinical signs of anorexia, apathy, grinding of teeth, grunting, abdominal distension, ruminal stasis, watery diarrhea, ataxia, and recumbency were observed after the grain was offered to sheep. Systemic examinations were carried out before and after treatment [6, 11].

*(1) Pulse and Respiration Taking*. The pulse rate was taken at the femoral artery [13]. The fingers were put on the femoral artery and gentle pressure was applied until the wave was determined. The respiration rate was counted by numbering the movements of the rib cage and abdomen [14].

*(2) Temperature Taking*. The body temperature was measured using a digital clinical thermometer. The digital thermometer was gently inserted into the rectum. It was kept in the rectum for two minutes [15, 16].

#### 2.5.2. Observation of Ruminal Fluid

*(1) Ruminal Fluid pH*. Rumenocentesis was done to collect ruminal fluid by using 16 gauge needles with a disposable syringe. The penetration site was the ventral rumen on the left side five cm posterior to the last rib. The procedure was done based on the standard [17]. The ruminal fluid was used for the evaluation of ruminal pH, which was measured using pH indicator paper or pH meter [18]. The ruminal fluid was placed on the paper and pH was observed by changing the color of the litmus paper and differentiating it from the standard colors of the indicator paper. The ruminal fluid was placed in a beaker and then a pH meter was inserted into it. So, the pH value was evaluated as the mean value [19].

*(2) Protozoa Activity Test*. A drop of ruminal fluid was put on a glass slide. The coverslip was placed on it. Then, it was observed under a low-power microscope for the presence or absence of ruminal protozoa. Protozoan motility was assessed in four groups: highly motile (++++) and very confined (good): >10 movable protozoa per field; motile (+++) and confined (fair): 6–9 movable protozoa per field; retarded movability (++) and few numbers (subnormal): 3–5 motile protozoa per field; + no or sporadic alive fauna (very low): <3 motile protozoa per field [20].

#### 2.5.3. Assessing of Hematological Parameter

*(1). Blood Sample Collection*. Blood sample collection was sampled from the jugular vein containing five ml with tubes without anticoagulant and tubes containing EDTA (ethylene diamine tetra acetic acid). Total white blood cell count (TWBC), total red blood cell (TRBC), hemoglobin estimation (Hb %), and packed cell volume (PCV) were evaluated by using an automatic haem-analyzer. The hematological evaluation was done through an automated hematology analyzer [16].

*(2) Determination of Blood pH*. A blood sample was taken from the jugular vein by using a test tube containing 5 ml. The sample was permitted to clot at room temperature for one hour to obtain serum [17]. The serum pH was evaluated by using pH indicator paper and pH meter [20]. The pH indicator paper was inserted into serum and the color of the strip was differentiated from the standard colors [18]. The serum was put into a beaker and a pH meter was inserted into it. The mean value of both readings was evaluated [21].

#### 2.5.4. Clinical Trials

Clinical trials were carried out to differentiate the best remedial agents. *Bicart-*sodium bicarbonate, digestive powder, and Magnalax-magnesium hydroxide treatment options were used for ruminal lactic acidosis as indicated in Table 1. The remedial agents were offered randomly to each group. Group I animals were given baking soda at a dose of 1 g/kg body weight. Group II was given Magnalax-magnesium hydroxide at a dose of 1.11 g/kg body weight. Animals in group III were treated with digestive powder at a dose of 0.8 g/kg. The remaining sheep were considered as negative control (group IV). Sampling was performed before the onset of therapy and thereafter on post-treatment [21, 23].

The therapeutic effectiveness was evaluated based on the disappearance of clinical signs, change of disturbed vital signs, ruminal pH, and hematological variable to the normal range [24]. It was also evaluated by the survival rate. Finally, the accuracy of the remedial agent was assured based on the clinical recovery of the sheep and rumen function tests [23].

### 2.6. Data Analysis

Data were collected and entered into an MS Excel spreadsheet. Then, it was processed using Stata MP 17. The data were analyzed using ANOVA and *t*-test. The hematological variable, ruminal, and clinical variables before and after therapy were calculated by one-way ANOVA. The acidotic groups were differentiated before and after therapy from the negative control group by paired *t*-tests. *P* value <0.05 was considered as statistically significant.

## 3. Results

### 3.1. Physical Parameter

The summary of clinical variables results is described in Table 2. The mean rectal temperature values recorded before treatment were 38.48 C° ± 0.20, 38.6 C° ± 0.21, 38.71 C° ± 0.54, and 39.32 C° ± 0.27 for groups I, II, III, and IV, respectively, as indicated in Table 2. The mean rectal temperatures calculated after treatment were 39.02 C°± 0.32, 39.11 C° ± 0.14, 39.23 C° ± 0.33, and 39.32 C° ± 0.27 for groups I, II, III, and IV, respectively. The mean pulse rate values before treatment for groups I, II, III, and IV were 123.5/min ±27.15, 115.5/min ±18.89, 117.25/min ±21.29 and 82/min ±6.19, respectively, as shown in Table 2. After treatment, the pulse rate of the groups I, II, III, and IV were again determined. The mean pulse rate values calculated after treatment were 93.5/min ±30.61, 88/min ±6.68, 86/min ±7.37, and 82/min ±6.19 for groups I, II, III, and IV, respectively. The mean skin turgor values counted before treatment were 3.37 ± 1.77, 4.12 ± 2.43, 3.12 ± 1.88 and 1 ± 0.00 second for groups I, II, III, and IV, respectively, as shown in Table 2. There was a significant (*p* < 0.05) increment in skin turgor of all groups of acidotic sheep as compared to healthy control. The mean skin turgor values of groups I, II, III, and IV after treatment were 2.25 ± 1.48, 2 ± 1.78, and 1.37 ± 0.86 and 1 ± 0.00 seconds, respectively.

### 3.2. Ruminal Fluid Examination

#### 3.2.1. Ruminal Fluid pH

The rumen pH mean values before treatment were 5.37 ± 0.34, 5.28 ± 0.58, 5.42 ± 0.48, and 6.65 ± 0.25 for groups I, II, III, and IV successively (Table 3). The rumen pH mean values observed after the remedial agent were 6.31 ± 0.43, 6.58 ± 0.30, 6.42 ± 0.37, and 6.65 ± 0.25 for groups I, II, III, and IV successively. The increase in ruminal fluid pH value after remedial agent in all groups of acidotic sheep was statistically significant (*p* < 0.05) as assessed to their pretreatment value.

#### 3.2.2. Protozoa Activity

Ruminal protozoa want appropriate pH for their survival. There was a significant (*p* < 0.05) reduction in protozoa motility of acidotic sheep as we differentiated to negative control. After the remedial agent, the protozoa activity test was carried out for all groups. The protozoa motility in group II was the lowest as we contrasted to groups I, III, and IV as summarized in Table 3. The improvement in protozoa motility after medical treatment in all the groups of acidotic sheep was statistically significant (*p* < 0.05) as contrasted to their pretreatment value.

### 3.3. Hematological Variables

The summary of hematological variables is shown in Table 4. The average total erythrocyte count in ruminal acidotic sheep of groups I, II, and III was 14.05 ± 1.27, 14.08 ± 1.75, and 13.6 ± 1.46 over 11.32 ± 0.54 group IV before treatment. The average total erythrocyte count (x10^6^/µL) in acidotic sheep of groups I, II, III, and IV after treatment was 12.71 ± 1.99, 11.8 ± 0.92, 11.5 ± 0.85, and 11.32 ± 0.54, respectively, as described in Table 4. The total leukocyte count of ruminal acidotic sheep of groups I, II, and III was evident from values of 12.71 ± 0.99, 12.3 ± 1.82, and 11.83 ± 1.12, respectively, over 9.05 ± 0.80 of group IV before treatment. The increment in total leukocyte count in all three ruminal acidotic groups was highly significant (*p* < 0.05) as compared to the healthy control group. The total leukocyte count declined to 10.95 ± 1.73, 10.51 ± 1.20, 10.3 ± 0.89, and 9.05 ± 0.80 in groups I, II, III, and IV, respectively, after therapy in acidotic sheep as shown in Table 4. The average hematocrit (PCV) in ruminal acidotic sheep of groups I, II, and III were 40.78 ± 5.24, 40.5 ± 5.93, and 39.28 ± 20.86 over 29.41 ± 3.25 of group IV before treatment. The hematocrit values declined to 35.27 ± 6.24, 33.87 ± 3.78, 31.68 ± 2.71, and 29.41 ± 3.25 in groups I, II, III, and IV, respectively, after treatment as indicated in Table 4. The blood pH of all ruminal acidotic sheep before treatment was found to be lower than the blood pH of the healthy control group. The mean blood pH values recorded before treatment were 6.91 ± 0.44, 6.92 ± 0.24, 7.02 ± 0.24, and 7.26 ± 0.17 for groups I, II, III, and IV, respectively. The blood pH values obtained after treatment were 7.11 ± 0.28, 7.31 ± 0.18, 7.21 ± 0.20, and 7.26 ± 0.17 for groups I, II, III, and IV, respectively, as shown in Table 4.

### 3.4. Remedial Accuracy Determination

Treatment accuracy of the remedial agents was assured based on clinical recovery from systemic acidosis. The summary of treatment efficacy is illustrated in Table 5. When we compared the remedial agent efficacy, digestive powder was the first drug followed by Magnalax and baking soda was ranked at the end based on the clinical recovery of the sheep.

## 4. Discussion

### 4.1. Vital Sign Parameter

The reduction of body temperature observed in the current study had similar findings to [25], which was due to fluid and electrolyte loss [4]. The increment of pulse rate was due to metabolic acidosis activation of the sympathetic nervous system [26, 27]. The increased respiration rate noted in the current study had similar findings to [28, 29]. The elevation of respiration rate above normal level was due to activation of medulla oblongata because of increased carbon-dioxide tension of blood and reduced blood pH [27, 30].

The occurrence of hypomotility or muscle atony during grain overload was due to the deactivation of the sympathomimetic ganglion [31]. The elevation of CRT and skin turgor in acidotic sheep had similar findings [32, 33]. This is due to profuse diarrhea [27].

### 4.2. Ruminal Fluid Examination

The reduction in pH of the ruminal fluid was observed in the current study due to the increasing production of volatile fatty acids. Ingestion of fermentable carbohydrates leads to a change in the microflora in the rumen within 2–6 hours [29]. The *Streptococcus bovis* increases in number rapidly, which leads to the production high amount of lactic acid. When the rumen pH is below five, it kills the microflora of the rumen. Lactobacilli bacteria ferment again the carbohydrate and induce a high amount of lactic acid [33, 34]. The superimposition of lactic acid and lactate in the rumen liquid results in the movement of fluid into the rumen and so, leads to dehydration. The present study had similar findings with [11, 18, 25].

The activity of the ruminal protozoa depends on the H^+^ ion and osmolality of the rumen liquor. In the present study, ruminal protozoa motility in acidosis sheep was usually absent which had a similar report from [35]. Magnalax treatment increased the rumen pH, but it reduced rumen protozoa motility. This finding was also reported by [30, 36, 37].

### 4.3. Hematological Parameters

The elevation of TRBC, TWBC, PCV, and Hgb in acidotic sheep was observed similar to [36, 38]. This is due to fluid and electrolyte loss because of profuse diarrhea [39]. The increment in TRBC in lactic acidotic sheep was due to the stress effect of systemic acidosis and epinephrine releasement causing splenic contraction [40, 41]. The current findings of increased TRBC in ruminal acidotic sheep are in agreement with [37].

The elevation of TWBC in ruminal acidosis is due to the production of endotoxins [34, 42]. This finding was also reported by [38].

Elevation of PCV in systemic acidosis sheep was due to hem concentration and dehydration, which was also reported by [41].

The elevation of hemoglobin concentration in the current study in lactic acidotic sheep had similar findings with [4, 30, 40]. The clinical recovery and systemic changes after treatment have occurred, which were also reported by [32, 43].

The blood pH of ruminal lactic acidotic sheep in the present study was below the normal value, which was due to lactate absorption from the rumen. This report had similarities with [44]. The elevation of blood pH after medicinal treatment was due to the buffering or alkalinizing effect of the remedial agent as in agreement with [42, 45].

### 4.4. Remedial Efficacy Assessment

In the current study, baking soda, Magnalax, and digestive powder were applied as remedies for ruminal lactic acidosis. All remedial agents were able to treat the acidotic condition as described in Table 5. When we compared the remedial agent efficacy, digestive powder was the first drug, followed by Magnalax and baking soda was ranked at the end based on the clinical recovery of the sheep. This showed that digestive powder was the most effective remedial agent which is in close agreement with [39, 45].

## 5. Conclusion and Recommendations

Ruminal lactic acidosis is a metabolic disorder defined by decreased blood pH and bicarbonate, caused by overproduction of ruminal D-lactate. The present clinical trial indicated that the acidosis sheep had lower body temperature, rumen motility, protozoa activity, and ruminal fluid pH. However, there was an elevation in pulse rate, respiration rate, CRT, and skin turgor. There were also significant (*p* < 0.05) increments in TEC, TLC, PCV, and Hgb in acidosis sheep. After the remedial agent application in acidotic sheep, the clinical and hematological parameters returned to normal levels. The current finding showed that all remedial agents were able to treat the acidotic condition. Digestive powder has various compositions, which are absorbed into the body and correct the disturbed clinical and hematological parameters. Digestive powder was the drug of choice, followed by Magnalax and baking soda was ranked at the end based on clinical and systemic recovery. In addition to this, sheep should be introduced gradually to concentrate rations over 2–3 weeks with a mixture of concentrate in the milled feed containing roughage.

Based on the above conclusions the following recommendations are forwarded:

- We shall avoid sudden feed change from roughage to concentrate to reduce the occurrence of ruminal lactic acidosis
- Digestive powder has various compositions and can correct hematological and clinical changes easily and rapidly
- Additional studies had better be performed about the cause of ruminal lactic acidosis in ruminants

## Figures and Tables

**Table 1:** Therapeutic trial for the treatment of ruminal acidosis in sheep.

Group	Number of sheep	Drugs	Composition	Dose (g/k.g)	Dosage (ml/k.g) and route	Frequency and duration of treatment
Group I (positive control)	8	Baking soda	Chloride, lead, calcium, iron, and arsenic	1	10 orally	Once a day for 4 days
Group II (Pc)	8	Magnalax	MgOH (Mg^+^, OH^−^)	1.11	10 orally	Once a day for 4 days
Group III (Pc)	8	Digestive powder	Sodium propionate 37.5 g, paracetamol 12.5 g, glucose monohydrate 12.5 g, and sodium hydrogen carbonate 37.5 g	0.8	8 orally	Twice a day for 4 days
Group IV (negative control)	8	Without treatment	Without treatment	Untreated	Untreated	No treatment

Source: [22].

**Table 2:** Clinical variables for a variety of groups due to grain overload effect (values are mean ± SE).

Clinical variables		Group I	Group II	Group III	Group IV	*P* value
		Sodium bicarbonate	Magnesium hydroxide	Digestion powder	No treatment	
Temperature (C°)	Before therapy	38.48^*∗*^ ± 0.20	38.6^*∗*^ ± 0.21	38.71^*∗*^ ± 0.54	39.32 ± 0.27	0.0002
	After therapy	39.02 ± 0.32	39.11 ± 0.14	39.23 ± 0.33	39.32 ± 0.27	0.0063

Pulse rate/min	Before therapy	123.5^*∗*^ ± 27.15	115.5^*∗*^ ± 18.89	117.25^*∗*^ ± 21.29	82 ± 6.19	0.001
	After therapy	93.5 ± 30.61	88 ± 6.68	86 ± 7.37	82 ± 6.19	0.1596

Respiratory rate/min	Before therapy	73.75^*∗*^ ± 12.71	60.5^*∗*^ ± 13.93	57^*∗*^ ± 12.51	36.75 ± 6.67	0.001
	After t	44 ± 25.21	40 ± 4.37	37.5 ± 3.89	36.75 ± 6.67	0.2822

Rumen motility/2 min	Before therapy	0.5^*∗*^ ± 0.89	0.37^*∗*^ ± 0.86	0.62^*∗*^ ± 1.24	2.5 ± 1.54	0.001
	After therapy	2.12 ± 1.65	1.75 ± 1.18	2.37 ± 1.24	2.5 ± 1.54	0.3244

Capillary refill time in sec	Before therapy	3^*∗*^ ± 1.78	2.87^*∗*^ ± 0.69	2.12^*∗*^ ± 1.39	1 ± 0.00	0.00001
	After therapy	1.75 ± 1.73	1.5 ± 0.44	1.12 ± 0.59	1 ± 0.00	0.0752

Skin turgor in sec	Before therapy	3.37^*∗*^ ± 1.77	4.12^*∗*^ ± 2.43	3.12^*∗*^ ± 1.88	1 ± 0.00	0.001
	After therapy	2.251 ± 1.48	2 ± 1.78	1.37 ± 0.86	1 ± 0.00	0.00083

^*∗*^Superscripts indicate statistical significance at *p* < 0.05 as it differentiated to group IV.

**Table 3:** Ruminal fluid analysis was performed in indigenous sheep breed to check protozoal activity (values are mean ± SE).

Ruminal fluid Analysis			Group I	Group II	Group III	Group IV
			Sodium bicarbonate	Magnesium hydroxide	Digestion powder	No treatment
Rumen PH	Before therapy		5.37^*∗*^ ± 0.34	5.28^*∗*^ ± 0.58	5.42^*∗*^ ± 0.48	6.65 ± 0.25
	After therapy		6.31 ± 0.43	6.58 ± 0.30	6.42 ± 0.37	6.65 ± 0.25

Protozoa activity	Before therapy	Absent	6 (75%)	7 (87.5%)	5 (62.5%)	0%
		Mild	2 (25%)	1 (12.5%)	3 (37.5%)	1 (12.5%)
		Moderate	0%	0%	0%	3 (37.5%)
		High	0%	0%	0%	4 (50%)
	After therapy	Absent	1 (12.5%)	4 (50%)	0%	0%
		Mild	4 (50%)	3 (37.5%)	4 (50%)	1 (12.5%)
		Moderate	3 (37.5%)	1 (12.5%)	3 (37.5%)	3 (37.5%)
		High	0%	0%	1 (12.5%)	4 (50%)

^*∗*^Superscripts describe statistical significance at *p* < 0.05 as it contrasted to group IV.

**Table 4:** Hematological variables change in indigenous sheep breed due to grain overload (values are mean ± SE).

Hematological variables		Group I	Group II	Group III	Group IV	*P* value
		Sodium bicarbonate	Magnesium hydroxide	Digestion powder	No treatment	
TRBC (x10^6^/*µ*L)	Before therapy	14.05^*∗*^ ± 1.27	14.08^*∗*^ ± 1.75	13.6^*∗*^ ± 1.46	11.32 ± 0.54	0.001
	After therapy	12.71 ± 1.99	11.8 ± 0.92	11.5 ± 0.85	11.32 ± 0.54	0.0032

TWBC (x10^3^/*µ*L)	Before therapy	12.71^*∗*^ ± 0.99	12.3^*∗*^ ± 1.82	11.83^*∗*^ ± 1.12	9.05 ± 0.80	0.001
	After therapy	10.95 ± 1.73	10.51 ± 1.20	10.3 ± 0.89	9.05 ± 0.80	0.0001

PCV (%)	Before therapy	40.78^*∗*^ ± 5.24	40.5^*∗*^ ± 5.93	34.78^*∗*^ ± 20.86	29.41 ± 3.25	0.0059
	After therapy	35.27 ± 6.24	33.87 ± 3.78	31.68 ± 2.71	29.41 ± 3.25	0.0004

Hgb (g/dL)	Before therapy	14.78^*∗*^ ± 1.83	14.61^*∗*^ ± 2.24	14.03^*∗*^ ± 1.73	10.97 ± 0.66	0.001
	After therapy	11.87 ± 2.68	11.48 ± 1.20	11.2 ± 0.66	10.97 ± 0.66	0.2558

Blood pH	Before therapy	6.91^*∗*^ ± 0.44	6.92^*∗*^ ± 0.24	7.02^*∗*^ ± 0.24	7.26 ± 0.17	0.0015
	After therapy	7.11 ± 0.28	7.31 ± 0.2	7.21 ± 0.20	7.26 ± 0.17	0.0324

^*∗*^Superscripts indicate statistical significance at *p* < 0.05 as it contrasted to group IV.

**Table 5:** Remedial effectiveness of various therapeutic regimens in ruminal lactic acidosis in sheep.

Group	Remedial agent	No. of sheep	Remedial effectiveness (% recovery)			
			Day 1	Day 2	Day 3	Day 4
Group I	Baking soda	8	2 (25%)	4 (50%)	6 (75%)	7 (87.5%)
Group II	Magnalax	8	4 (50%)	5 (62.5%)	7 (87.5%)	8 (100%)
Group III	Digestive powder	8	5 (62.5%)	7 (87.5%)	8 (100%)	8 (100%)

## Data Availability

The data that support the findings of this study are available from the corresponding author upon reasonable request.

## Abbreviations

CRT: Capillary refill time
CSA: Central statistical agency
EDTA: Ethylene diamine tetra acetic acid
LRS: Lactated ringer solution
PCV: Packed cell volume
PME: Polioencephalomalacia
RFC: Readily fermentable carbohydrate
SARA: Subacute ruminal acidosis
TEC: Total erythrocyte count
VFA: Volatile fatty acids.

## References

[1] Khaskheli A. A., Khaskheli M. I., Khaskheli A. J., Khaskheli A. A. (2020). Aceh journal of animal science A mini review on the lactic acidosis in goats and its remedial approaches. *Aceh Journal of Animal Science*.

[2] Sabes A. F., Girardi A. M., Filho D. Z., Bueno G. M., Oliveira J. A., Marques L. C. (2017). Acid-base balance in sheep with experimentally induced acute ruminal lactic acidosis. *Arquivo Brasileiro de Medicina Veterinária e Zootecnia*.

[3] Sabes A. F., Girardi A. M., Fagliari J. J., De Oliveira J. A., Marques L. C. (2017). Serum proteinogram in sheep with acute ruminal lactic acidosis. *International Journal of Veterinary Science and Medicine*.

[4] Mahmood A. K., Khan M. S., Khan M. A., Khan M. A., Bilal M., Farooq U. (2013). Lactic acidosis in goats: prevalence, intra-ruminal and haematological investigations. *Journal of Animal and Plant Sciences*.

[5] Kirbas A., Baydar E., Kandemir F. M., Dorman E., Kizil O., Yildirim B. A. (2014). Evaluation of serum cardiac troponin I concentration in sheep with acute ruminal lactic acidosis. *Veterinarski Arhiv*.

[6] P A. (2016). Effect of sub-acute ruminal acidosis (SARA) on milk quality and production performances in commercial dairy farms. *Journal of Livestock Science*.

[7] Anderson D. P., Jeffcott L. B., Quesenberry K. E., Radostits O. M., Reeves P. T., Wolf A. M. (2005). *The Merck Veterinary Manual*.

[8] Nikolov Y., Angelov A. (1996). Changes in acid-base parameters, blood sugar, and blood lactate in experimental acute rumen acidosis in sheep. *Indian Veterinary Journal*.

[9] A V., Thamsborg S. M., Jorgensen R. J., Basse A. (1995). Induced acute ruminal acidosis in goats treated with yeast (*Saccharomyces cerevisiae*) and bicarbonate. *Acta Veterinaria Scandinavica*.

[10] Achenef M., Shimelis D. (2022). Therapeutic efficacy trial for different treatment regimens due to induced ruminal acidosis in sheep in Addis zemen town, Ethiopia. *Research Square*.

[11] South Gondar Zone Administration Central Statistical Agency (Sgacsa) (2018). *Agricultural Office of Livestock and Livestock Characteristics*.

[12] Muralidhara A., Ravindranath B. M. (2011). Ruminal acidosis in small ruminants and its therapeutic management. *Journal of Animal Science*.

[13] Hinchcliff S. H., Grunberg W. (2017). *A Textbook of Diseases of Cattle, Horse, Sheep, Pigs, and Goats*.

[14] Suresh R. V., Thangathurai R., Dhanapalan (2017). Retrospective study on ruminal acidosis in goats. *Indian Veterinary Journal*.

[15] Deboever D. D. L., Vanacker j. l., and Geerts j. M. (2002). *Evaluation and Effects of Physical Structure in Dairy Cattle Nutrition*.

[16] Han G., Gao X., Duan J. (2021). Effects of yeasts on rumen bacterial flora, abnormal metabolites, and blood gas in sheep with induced subacute ruminal acidosis. *Animal Feed Science and Technology*.

[17] Hammond P. B. (1995). D-Lactic acidosis of ruminants. *Academic Science Journal*.

[18] Sconza S., Lorenz I., Otranto G., Rademacher G., Klee W. (2004). D-lactic acidosis in calves as a consequence of experimentally induced ruminal acidosis. *Journal of Veterinary Clinics*.

[19] Jaramillo-lópez E., Itza-ortiz M. F., Peraza-mercado G., Carrera-Chávez J. M. (2017). Ruminal acidosis: strategies for its control. *Austral Journal of Veterinary Sciences*.

[20] G N (2006). Effect of supplemental yeast culture and Sodium bicarbonate on Ruminal fermentation and blood variables in rams. *Journal of Animal Physiology and Animal Nutrition*.

[21] Yadav R., Sharma C. S., Gattani A. (2012). Dietary induced metabolic acidosis in goats and its successful therapeutic management. *Veterinary Practitioner Bikaner*.

[22] H H. R., Nouri M., Afshar F. S., Dehkordi A. (2006). Effects of experimentally induced ruminal acidosis on blood pH, bicarbonate and CO_2_ in the sheep. *Pakistan Journal of Biological Sciences*.

[23] Hernández J., Benedito J. L., Abuelo A., Castillo C. (2014). Ruminal acidosis in feedlot: from aetiology to prevention. *The Scientific World Journal*.

[24] RidLorenz I., Gentile A. (2014). D-lactic acidosis in neonatal ruminants. *Veterinary Clinics of North America: Food Animal Practice*.

[25] K J. A., Khan M. S., Sadique U., Shah M., Idrees M., Shah Z. (2013). ClinicoTherapeutical trials of lactic acidosis in small ruminants. *Journal of Animal and Plant Sciences*.

[26] Bertoni G., Jahan N., Bani P. (2018). Experimental Acute Rumen Acidosis in Sheep: consequences on rumen and gastrointestinal permeability conditions and blood chemistry. *American Society of Animal Science*.

[27] Nour M. S. M., Abusamra M. T., Hago B. D. (1998). Experimentally induced lactic acidosis in nubian goats: clinical, biochemical and pathological investigations. *Small Ruminant Research*.

[28] Abdel I. A., Baraka T. A. (2010). Influence of rumen acidosis on clinical, hematological and biochemical constituents of blood and rumen liquor in Egyptian dairy cattle. *International Congress of Mediterranean Federation of Health Aussiut*.

[29] Nambi A. P., Vijaykumar G., Vairamuthu S. (2012). Efficacy of hemodialysis in goats affected with acute ruminal acidosis. *Indian Veterinary Journal*.

[30] Gay C. C., Hinchcliff K. W., Constable P. D. (2007). *Textbook of Diseases of Cattle, Horses, Sheep, and Goats*.

[31] P M N (2013). Clinicopathological and therapeutic studies in acute ruminal acidosis of goats. *Submitted MVSc Thesis to Karnataka*.

[32] P K. R., Verma S. P., Anup K. A., Jayachandran C. (2007). Effect of severity of acidosis on ruminal activity in goats. *Indian Journal of Animal Sciences*.

[33] Sarma S., Nath R. (2005). Studies on rumen acidosis in goat and efficacy of treatment. *Journal of Veterinary Medicine A*.

[34] S G. W., Correa M. T. (2004). The effects of oral Magnesium hydroxide administration on rumen fluid in Cattle. *Journal of Veterinary Internal Medicine*.

[35] Gay C. C., Blood D. C., Hinchcliff K. W. (2000). *Veterinary Medicine*.

[36] Shaheen G., Gupta A., Lather S., Nabi A. R., Hassan M. (2013). Clinical and haematological changes in rumen acidosis in south down breed of sheep in kashmir valley. *The Haryana Veterinarian*.

[37] Pillai U. N., Ajitkumar S. (2006). Serum biochemical, physico-chemical and microbiological changes in rumen liquor of experimental ruminal acidosis in goats. *Indian Journal of Veterinary Medicine*.

[38] Padmaja N. A., Gopala R. (2017). Studies on rumen fluid analysis in ruminal acidotic goats. *International Journal of Livestock Research*.

[39] Hussain T., Amin U., Rouf A., Dar Z. A., Nabi S. U., Shaheen M. (2017). Efficacy of different therapeutic regimens for simple indigestion in sheep. *Journal of Animal Science*.

[40] Usha N. P., Ajithkumar S., Alex P. C. (2003). Haematological change in experimental luminal acidosis in goats. *Indian Journal of Veterinary Medicine*.

[41] T M (2013). Rumen acidosis in small ruminants and its therapeutic management. *Iranian Journal of Applied Animal Science*.

[42] V T. S., Padmaja K., Nagaraj P., Gopala A. (2017). Therapeutic studies of ruminal acidosis in goats. *Journal of Pharmaceutical Innovation*.

[43] Camera A. C., Mendonce C. L., Afonso A. B. (2012). Haematological and biochemical profile of sheep supplemented with salinomycin and submitted to experimental ruminal lactic acidosis. *Journal of Animal Health and Production*.

[44] B W. (2006). Grain poisoning of cattle and sheep. *Journal of Animal Health*.

[45] Z M. M., Ghanem M. M., Abdraof Y. M., Eiattar H. M., Eikhaiat M. (2014). Clinical, Haemato-biochemical and ruminal changes during the onset and recovery of induced Lacto Acidosis in Sheep. *Biotechnology in Animal Husbandry*.

